# A Molecular Link between Malaria and Epstein–Barr Virus Reactivation

**DOI:** 10.1371/journal.ppat.0030080

**Published:** 2007-06-08

**Authors:** Arnaud Chêne, Daria Donati, André Ortlieb Guerreiro-Cacais, Victor Levitsky, Qijun Chen, Kerstin I Falk, Jackson Orem, Fred Kironde, Mats Wahlgren, Maria Teresa Bejarano

**Affiliations:** 1 Center for Infectious Medicine, Department of Medicine, Karolinska Institutet, Stockholm, Sweden; 2 Department of Microbiology, Tumor and Cell Biology, Karolinska Institutet, Stockholm, Sweden; 3 Swedish Institute for Infectious Disease Control, Stockholm, Sweden; 4 Uganda Cancer Institute, Kampala, Uganda; 5 Department of Biochemistry, Faculty of Medicine, Makerere University, Kampala, Uganda; University of Wisconsin–Madison, United States of America

## Abstract

Although malaria and Epstein–Barr (EBV) infection are recognized cofactors in the genesis of endemic Burkitt lymphoma (BL), their relative contribution is not understood. BL, the most common paediatric cancer in equatorial Africa, is a high-grade B cell lymphoma characterized by *c-myc* translocation. EBV is a ubiquitous B lymphotropic virus that persists in a latent state after primary infection, and in Africa, most children have sero-converted by 3 y of age. Malaria infection profoundly affects the B cell compartment, inducing polyclonal activation and hyper-gammaglobulinemia. We recently identified the cystein-rich inter-domain region 1α (CIDR1α) of the Plasmodium falciparum membrane protein 1 as a polyclonal B cell activator that preferentially activates the memory compartment, where EBV is known to persist. Here, we have addressed the mechanisms of interaction between CIDR1α and EBV in the context of B cells. We show that CIDR1α binds to the EBV-positive B cell line Akata and increases the number of cells switching to the viral lytic cycle as measured by green fluorescent protein (GFP) expression driven by a lytic promoter. The virus production in CIDR1α-exposed cultures was directly proportional to the number of GFP-positive Akata cells (lytic EBV) and to the increased expression of the EBV lytic promoter BZLF1. Furthermore, CIDR1α stimulated the production of EBV in peripheral blood mononuclear cells derived from healthy donors and children with BL. Our results suggest that P. falciparum antigens such as CIDR1α can directly induce EBV reactivation during malaria infection that may increase the risk of BL development for children living in malaria-endemic areas. To our knowledge, this is the first report to show that a microbial protein can drive a latently infected B cell into EBV replication.

## Author Summary

Malaria and Epstein–Barr virus (EBV) infections are recognized cofactors in the genesis of endemic Burkitt lymphoma, the most common paediatric cancer in equatorial Africa. EBV is a ubiquitous virus residing in B lymphocytes that establishes a lifelong persistence in the host after primary infection. EBV has two lifestyles: latent infection (non-productive), and lytic replication (productive). Children living in malaria-endemic areas exhibit an elevated viral load, and acute malaria infection increases the levels of circulating EBV. The mechanisms leading to viral reactivation during Plasmodium falciparum malaria infection are not well understood. Cystein-rich inter-domain region 1α (CIDR1α) is a domain of a large protein expressed at the surface of P. falciparum–infected red blood cells. Based on previous findings showing that CIDR1α activates and expands the B cells compartment where EBV persists, we assessed the impact of CIDR1α on viral reactivation. Here, we identify CIDR1α as the first microbial protein able to drive a latently EBV-infected B cell (no virus production) into lytic replication (virus production). Our results suggest that P. falciparum–derived proteins can lead to a direct reactivation of EBV during acute malaria infection, increasing the risk of Burkitt lymphoma development for children living in malaria-endemic areas.

## Introduction

Epstein–Barr virus (EBV) is a human γ-herpes virus that establishes a persistent infection in >90% of the world's population. Like other herpes viruses, EBV has two alternative lifestyles: latent (non-productive) infection, and lytic (productive) replication. Following primary infection, EBV persists within memory B lymphocytes in a latent state for the life of the host. A low level of reactivation into lytic replication allows viral shedding into the saliva and transmission of the virus in vivo [1]. The lifelong persistent infection established by EBV is harmless in almost every host and rarely causes disease, unless the host–virus equilibrium is upset. Thus, viral persistence represents a balance between viral latency, viral replication, and host immune responses.

The lytic phase of viral replication can be triggered in vitro by a variety of reagents and stimuli, including halogenated pyrimidine [2], phorbol esters [3], calcium ionophores [4], transforming growth factor β [5], butyrate [6], and triggering of the B cell receptor (BCR) with anti-immunoglobulin (anti-Ig) antibody (Ab) [7]. Less is known about the physiological stimuli that control activation of the virus productive cycle in vivo, although replication seems to occur following plasma cell differentiation [8].

It has been well documented that EBV is causally associated with various malignancies, including endemic Burkitt lymphoma (BL), nasopharyngeal carcinoma, and B cell lymphoma, in immunocompromised hosts [9]. Both EBV infection and intense exposure to Plasmodium falciparum malaria (holoendemic malaria) are recognized cofactors in the pathogenesis of BL, which is the most common paediatric cancer in equatorial Africa, accounting for up to 74% of childhood malignant disorders [10]. Development of BL, a B cell malignancy, is heralded by high Ab titers to replicative antigens indicative of EBV reactivation [11]. Recent reports indicate that the impact of malaria infection on EBV persistence is reflected by an increased viral replication. Children living in malaria-endemic areas have an elevated EBV load [12,13], and acute malaria infection leads to increased levels of circulating EBV that are cleared following anti-malaria treatment [14].

The mechanisms that may lead to viral reactivation during P. falciparum malaria are not well understood. The identification of a polyclonal B cell activator and Ig-binding protein in P. falciparum is of particular relevance in this context. We demonstrated that the cystein-rich inter-domain region 1α (CIDR1α) of the *P. falciparum* erythrocyte membrane protein 1 (PfEMP1) induces proliferation and activation of B cells, preferentially of the memory subset, where EBV is known to reside [15,16]. To understand the relative contribution of malarial antigens on EBV reactivation, we used the prototype EBV-positive BL cell line Akata as a model to determine whether CIDR1α could induce reactivation of the EBV lytic cycle in latently infected B cells. Furthermore, we analyzed the effect of the CIDR1α on freshly isolated peripheral blood mononuclear cells (PBMCs) from EBV-positive healthy donors and from children with BL living in malaria-endemic areas. The results support the hypothesis that CIDR1α is one of the molecules involved in EBV reactivation during the course of malaria infection. Our data provide new insights into how malaria infection may contribute to BL development.

## Results

### P. falciparum–Infected Red Blood Cells and Purified CIDR1α Bind to EBV-Carrying B Cells

During the blood stage of P. falciparum malaria, infected red blood cells (iRBCs) express high levels of PfEMP1, reaching their maximum at the trophozoite stage (28–32 h post-invasion). The CIDR1α domain of PfEMP1 (clone FCR3S1.2-var1) has a multi-adhesive phenotype and binds to different cell surface receptors, such as CD36, PECAM-1 (CD31), and immunoglobulins (Igs) [17]. CIDR1α also binds to isolated B cells via an interaction that involves surface Igs [15].

**Figure 1:**
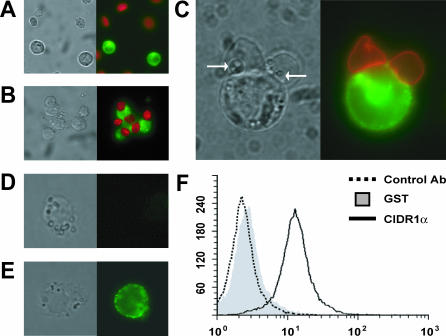
P. falciparum iRBCs and Soluble CIDR1α Bind to the EBV-Positive B Cell Line Akata (A–C) Akata cells stained with PKH67 (green) were co-incubated with RBCs stained with PK26 (red) (A) or with purified iRBCs (B, C) at a ratio of 1:2 (Akata:iRBC). Binding was evaluated by light microscopy (left panel) and immunofluorescence microscopy (right panel). (C) Magnification of the binding between iRBCs and Akatas. Localization of the parasite in the iRBC (indicated by white arrows) was revealed using phase contrast microscopy (left panel). (D–F) Akata cells were incubated with soluble GST (1 μM) (D) or CIDR1α (1 μM) (E) for 1 h at RT and stained with anti-GST Ab and anti-mouse Alexa-488-conjugated Ab. The binding was evaluated by light microscopy (left panel, [D, E]), immunofluorescence microscopy (right panel, [D, E]), and fluorescent-activated cell sorting (FACS) analysis (F).

To establish whether iRBCs and the soluble form of CIDR1α interact with EBV-carrying B cells, we used the EBV-positive BL cell line Akata as a model. Akata cells stained with PKH67 (green) were co-incubated with PKH26 (red)-stained uninfected red blood cells (RBCs), or with enriched iRBCs at the trophozoite stage (28 h post-invasion, 75%–80% final parasitemia), at a ratio of 1:2, respectively. RBCs did not bind to Akata (Figure 1A), but co-incubation with iRBCs led to the formation of conjugates that varied in size but frequently involved two to five iRBCs/Akata cell (Figure 1B). A higher magnification of the conjugates showed a polarization of the iRBC, where the parasites were found at the proximity of the membrane's tight junction between the two cell types (Figure 1C).

Being that CIDR1α is a domain expressed on iRBCs with multi-adhesive phenotypes [17], we investigated its ability to bind Akata cells. A soluble form of CIDR1α was produced as a glutathione-S-transferase (GST) fusion protein, and the GST protein alone was used as control. Immunofluorescence studies with anti-GST fluorescent Abs demonstrated that CIDR1α, but not the GST control, binds to the membrane of Akata cells (Figure 1D and 1E). Flow cytometry analysis showed a peak shift representing an increased mean fluorescence intensity (MFI) as compared to the MFI values obtained when Akata cells were incubated with GST control protein or with the isotype control Ab (Figure 1F). Thus, both iRBCs and the recombinant CIDR1α domain of PfEMP1 bind to the EBV-carrying B cell line Akata.

### CIDR1α and iRBC Stimulation Lead to Increased EBV Viral DNA Load in Akata Cells

In contrast to the variety of reagents and signals able to induce EBV lytic production in vitro [18], the physiological signals involved in the activation of the virus productive cycle have not been well characterized yet, although plasma cell differentiation seems to represent one such trigger [8]. Anti-Ig treatment, which leads to BCR signalling [19], has served as a relevant in vitro model of reactivation for inducing virus replication in some EBV-carrying BL cell lines, including Akata [7,20].

**Figure 2:**
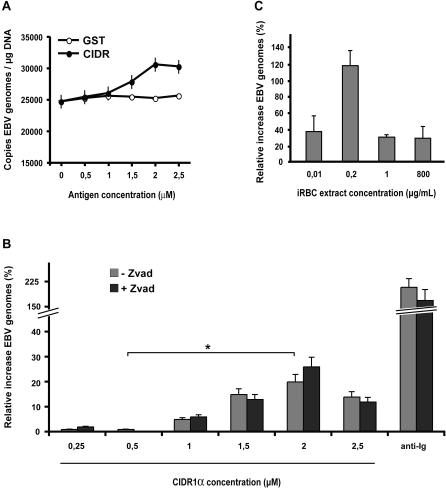
CIDR1α and iRBC Stimulation Lead to Increased Viral Load in Akata Cells (A) Akata cells were cultured in medium alone (referred to as Ag concentration 0) with GST (white circles) or with CIDR1α (black circles) at protein concentrations ranging from 0,5 to 2,5 μM in the presence or absence of zVAD. After 48 h of incubation, the viral genome copy number was determined by real-time PCR. Results from a representative experiment (out of four independent experiments) are expressed as numbers of EBV genome copies per μg of total DNA. (B) Akata cells were cultured with GST or CIDR1α (0–2,5 μM), and with anti-Ig (10 μg/mL). After 48 h of incubation, the viral genome copy number was determined by real-time PCR. Results are expressed as percentage of relative increase in EBV copy number in cultures stimulated with CIDR1α versus cultures stimulated with GST, and represent the mean of four independent experiments ± standard deviation. *, *p*_2vs0,5 μM _ = 0,03. (C) Akata cells were cultured with increasing concentrations of crude extracts from iRBCs and RBCs. After 48 h of incubation, the viral genome copy number was determined by real-time PCR. Results from one representative experiment (out of three performed) are expressed as percentage of relative increase in EBV copy numbers in cultures stimulated with iRBCs versus cultures stimulated with RBC extracts.

Because CIDR1α is an Ig-binding protein [17], we analyzed its functional impact on the reactivation of lytic virus production and used the well-characterized Akata cell line model as a read-out system. First, we analyzed whether stimulation of Akata cells with CIDR1α would affect the number of viral DNA copies produced. Cells were cultured with increasing concentrations of CIDR1α, GST (range 0,5–2 μM, corresponding to 25–100 μg/mL), or in medium alone. After 48 h, we quantified the EBV viral DNA copy number in the cultures (cells + supernatant) by monitoring the EBV LMP1 gene, which is present as a single copy in the virus genome. As shown in Figure 2A and 2B, stimulation with CIDR1α increased the viral DNA load in a dose-dependent manner. Cells incubated with GST contained numbers of EBV genomes comparable to that of cells cultured in medium alone. In two out of four independent experiments, there was a statistical significance in relative increase of EBV load between the concentrations of 0,5 μM and 2 μM (*p* = 0,03).

We have recently demonstrated that CIDR1α induces B cell proliferation and protects B cells from apoptosis [16]. It could be then argued that the augmented viral load might simply result from a net increase in the number of cells in the culture. Cell cycle analysis performed by propidium iodide staining after 24 and 48 h of incubation did not reveal any significant change in the proportion of dead (<G0/G1) or cycling cells (S-G2/M) between cultures containing CIDR1α, GST, or medium alone (unpublished data). Moreover, experiments carried out in the presence of z-VAD, a pan-specific caspase inhibitor that blocks apoptosis, did not significantly affect the extent of EBV DNA increase in cultures containing GST or CIDR1α (Figure 2B), ruling out the involvement of apoptosis. In conclusion, stimulation of the EBV-carrying B cell line Akata with CIDR1α leads to an increased number of viral genomes that is not dependent on apoptosis or increased proliferation.

The interaction between iRBCs and B cells is partially mediated by CIDR1α [15] that is expressed at the iRBC surface along with a variety of other antigens [21]. Therefore, it became of interest to see whether the interaction of Akata cells with iRBCs would lead to viral reactivation. Intact iRBCs could not be used for this purpose, as detection of viral production requires 48 h of co-incubation with Akata, and during this time the iRBCs burst, leading to toxicity and cell death. The erythrocytic parasite cycle from invasion to merozoite release takes 48 h and the purification of synchronized iRBCs requires a high cellular content of the paramagnetic pigment hemozoin that is reached 28 h post-invasion; i.e., the rupture would occur in the middle of the test. To overcome the iRBC burst and the related cytotoxicity, we used crude extracts from RBCs and iRBCs obtained 28 h post–parasite invasion. Incubation with iRBC extracts resulted in increased viral production as compared to exposure to RBC extracts (Figure 2C). The above results suggest that iRBC-derived molecules can induce viral production, although the role played by CIDR1α in this context is difficult to assess.

### The Increased Viral Copy Number Induced by CIDR1α Reflects Induction of the Lytic Cycle

To investigate whether the increased number of EBV genomes resulted from lytic cycle reactivation, we used an Akata cell line–based system in which induction of the lytic cycle is accompanied by increased expression of green fluorescent protein (GFP). Upon BCR cross-linking with anti-Ig, 20%–50% of cells typically enter the replicative cycle. Cells that support lytic replication up-regulate GFP (10- to 100-fold), whereas cells that remain in latency express little GFP. The kinetics of GFP expression follows that of early lytic genes. The GFP expression persists throughout the lytic cycle [22], and is therefore used as a quick and simple read-out for viral replication.

**Figure 3:**
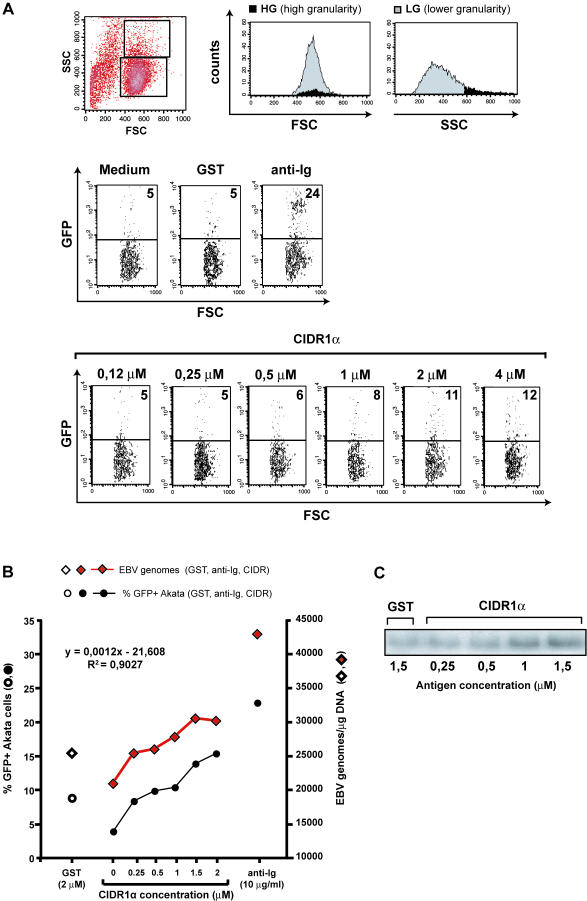
Induction of EBV Lytic Cycle in Akata-GFP Cells upon Stimulation with CIDR1α (A) Akata-GFP cells were cultured in medium alone, with GST (4 μM), with CIDR1α (0,12–4 μM), or with anti-Ig (10 μg/mL). After 48 h of incubation, a FACS analysis was performed. Cell populations were divided into cells with low granularity (LG) and cells with high granularity (HG) on the basis of side scatter (SSC). The proportion of cells in lytic cycle (GFP-positive) was then quantified in the HG subpopulation. The figure shows one representative experiment. FSC, forward scatter. (B) Akata-GFP cells were cultured with GST (2 μM), with CIDR1α (0–2 μM), and with anti-Ig (10 μg/mL). After 48 h of incubation, the proportion of GFP-positive cells was quantified by FACS analysis (circles), and the EBV DNA load was assessed by real-time PCR (diamonds). The left *y*-axis denotes the proportion of Akata-GFP–positive cells in lytic cycle, the right *y*-axis denotes the number of EBV genomes. Controls included GST-stimulated (empty diamonds/circles) and anti-Ig–stimulated cells (filled diamonds/circles). Data show the mean of three independent experiments. (C) Akata cells up-regulate BZLF1 upon stimulation with CIDR1α. Akata-GFP cells were cultured with GST (1,5 μM) or with CIDR1α (0,25–1,5 μM). After 48 h of incubation, the expression of BZLF1 was analyzed by Western blot. Increased BZLF1 expression in CIDR1α stimulated samples was confirmed by relative quantitative analysis with Quantity One software (Bio-Rad).

Akata-GFP cells were incubated with increasing concentrations of CIDR1α and GST for 48 h, at which time the extent of cells expressing GFP was measured by flow cytometry. Cells induced to lytic replication, either with CIDR1α or anti-Ig stimulation, and therefore expressing high levels of GFP, had a higher granularity (side scatter) and a minor increase in size (forward scatter) as compared to the GFP-negative population. These cellular changes reflect an activated status. Analysis of the data, gating on the region containing highly granular cells, showed that the increase in GFP-positive cells was 1,4 to 2,4 times higher among the CIDR1α-stimulated cells than in the GST control cultures. This is accounted by 12%–19% of cells in replication when cultured with 1 μM CIDR1α versus 5%–9% for cells cultured with GST (Figure 3A).

The increased proportion of cells in lytic cycle (GFP-positive) induced by CIDR1α treatment correlated with a rise in the number of viral genomes as measured by quantitative PCR. The rise in the proportion of GFP-positive cells after CIDR1α stimulation was directly proportional to the increase of EBV load (R^2^ = 0.90; *p* < 0,001) (Figure 3B).

Activation of lytic production by CIDR1α was further confirmed by analysis of the BZLF1 protein (also known as Z, Zebra, Zta, EB1) expression. BZLF1, an immediate early protein which acts as a transcriptional activator and disrupts EBV latency, is essential for full expression of lytic genes and viral DNA replication [23]. The kinetics of BZLF1 expression vary according to the infected cell type and the replication-inducing agent used. As early as 6 h after induction by surface Ig cross-linking, Akata cells start expressing BZLF1 and continue doing so throughout the lytic cycle [22]. Stimulation with CIDR1α led to an increased expression of BZLF1 in a CIDR1α dose-dependent manner as revealed by Western blot analysis 48 h after stimulation (Figure 3C). These results support the hypothesis that CIDR1α activation induces viral reactivation in EBV-carrying B cells. Taken together, the data indicate that the increased viral load induced by CIDR1α in Akata cells results from a switch in EBV from latency to the replicative lytic cycle.

### CIDR1α Stimulation Increases the Viral Copy Number in B Cells Derived from EBV-Positive Donors

B cells derived from the mucosal lymphoid tissue of the Waldeyer ring (tonsils) have a ten times higher frequency of virus-infected cells as compared to peripheral blood B cells [24]. To investigate the possible physiological impact that malaria infection has on EBV persistence, we analyzed the effect of CIDR1α on PBMCs and B cells derived from healthy EBV-positive donors with regard to quantitative changes in EBV DNA. PBMCs were co-incubated with CIDR1α, and after 48 h, the EBV genome copy number was quantified by real-time PCR and compared to the one in the GST/medium control cultures. This analysis was extended to PBMCs derived from children with BL living in malaria-endemic area.

**Figure 4:**
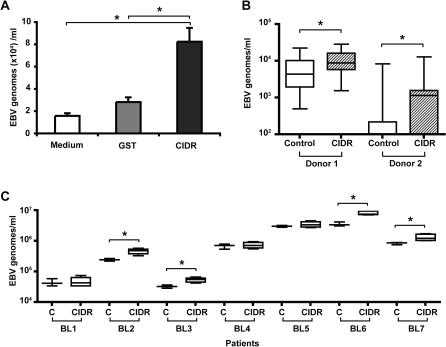
Stimulation of Tonsil B Cells and PBMCs from EBV-Positive Donors and BL Patients with CIDR1α Increases the EBV Load (A) Tonsil B cells were cultured in medium alone (white bar), with GST (1 μM) (light grey bar), and with CIDR1α (1 μM) (dark grey bar). After 48 h of incubation, the viral genome copy number in the cultures was determined by real-time PCR. *, *p* = 0,04. (B) PBMCs from two EBV-positive healthy donors were cultured with GST (1 μM) (white bars) and with CIDR1α (1 μM) (dashed bars). After 48 h of incubation, the viral genome copy number in the cultures was determined by real-time PCR. Data are expressed as EBV copies/mL. Vertical bars depict median values, with 25th and 75th percentile values represented by the bottom and top edges of the bar. Each bar shows the EBV load from one patient measured in multiple replicates and in three independent experiments. *, *p*_Donor1_ = 0,04; *, *p*_Donor2_ = 0,001. (C) PBMCs from children with BL were cultured with medium or GST (1 μM), our internal controls (C), and with CIDR1α (1 μM). After 48 h of incubation, the viral genome copy number in the cultures was determined by real-time PCR. Data are expressed as EBV copies/mL. Vertical bars depict median values, with 25th and 75th percentile values represented by the bottom and top edges of the bar. Each bar shows the EBV load measured in multiple replicates. *, *p*_BL2_ = 0,01; *, *p*_BL3_ = 0,01; *, *p*_BL6_ = 0,0003; *, *p*_BL7_ = 0,03.

Stimulation of tonsil B cells with CIDR1α increased by 3-fold the number of EBV genomes as compared to the control antigen (*p* = 0,04) (Figure 4A). In PBMCs, where the B cell number and the frequency of EBV-positive cells is lower, stimulation with CIDR1α led to a 1,4- and 2,5-fold increase in EBV genomes (Figure 4B). This increase was statistically significant (*p*_Donor1_ = 0,04; *p*_Donor2_ = 0,001). The two healthy donors used for this study have an elevated number of EBV-carrying B cells as reflected by a high frequency of spontaneous outgrowth of EBV-positive B cells when their PBMCs are cultured in vitro. The absolute levels of EBV genomes in PBMCs are lower than the ones in tonsil B cells, a fact that may be the result of a lower frequency of EBV-carrying B cells in PBMCs.

Although EBV infection is usually harmless, EBV is linked to several human cancers and it is recognized as a cofactor in the development of the endemic form of BL, in which virtually all tumour cells are EBV-positive. We have previously shown that the overall EBV DNA load is elevated in serum from children with BL [14]; therefore, it can be assumed that PBMCs from BL patients have a high frequency of EBV-positive B cells. Thus, we tested the effect of CIDR1α on PBMCs derived from seven Ugandan patients with BL. In four out of seven patients, stimulation with CIDR1α (2 μM) for 48 h resulted in an increased median viral load as compared to that of control cultures. The difference was statistically significant (*p* ≤ 0,03) (Figure 4C). Although the overall median value was comparable in CIDR1α- and GST-treated PBMCs in two of the three other patients, there were few replicate wells with higher EBV load compared to respective GST control (for both patients). Consequently, we conclude that CIDR1α stimulation increases the viral load in EBV-carrying B cells derived from EBV-positive healthy donors and in patients with BL.

## Discussion

Despite the well-established link between malaria and EBV infection with BL, little is known about the interaction between the two pathogens and the mechanisms responsible for the elevated EBV load observed in children living in malaria-endemic areas [12,25]. In this report, we identify, to our knowledge for the first time, a microbial protein from *P. falciparum,* that can drive a latently infected B cell into viral replication. Previous studies by Minoura-Etoh et al. demonstrated that monochloramine (NH_2_Cl), a Helicobacter pylori–associated oxidant, induces viral production in epithelial cells [26].

Following primary infection, EBV is kept latent within memory B cells. In vivo, terminal differentiation of B cells into plasma cells triggers the switch from latency into the lytic replicative cycle [8]. Signalling of BCR is initiated upon binding of the antigen to the membrane-bound Igs, and this activation contributes to cell differentiation and Ab production. The EBV reactivation in Akata cells that results from BCR cross-linking with anti-Ig may reflect physiological mechanisms that operate as latently infected memory B cells proceed through the germinal center reaction and/or undergo plasma cell differentiation. Given the fact that both CIDR1α and iRBCs bind non-immune Igs [15,27], it could be assumed that the induction of virus replication in Akata cells mediated by CIDR1α and by iRBC extracts may involve similar signalling pathways. We cannot ascertain that CIDR1α is the only molecule responsible for the viral production induced by iRBC crude extracts, because in addition to CIDR1α, iRBCs express other malarial antigens that could play a role in viral reactivation.

Children living in malaria-endemic areas have an elevated EBV load that is cleared after anti-malaria therapy [14] and is directly related to the level of endemicity [12]. The rise in viral load may partly be a consequence of the interaction between malarial antigens able to bind Igs and latently infected B lymphocytes. The interaction between B cells and iRBCs is of particular relevance; we have reported that non-immune B cells bind iRBCs, and that this interaction is partially mediated by CIDR1α on the iRBC membrane and Igs on the B cell surface [15]. Here, we show that the iRBCs and the malaria parasite protein CIDR1α bind to the EBV-positive B cell line Akata. Most likely, the Ig-binding capacity of CIDR1α [15] and potentially other P. falciparum antigens present on iRBCs could lead to viral replication in a manner similar to the way anti-Ig induces viral production in Akata cells. This assumption is based on the fact that CIDR1α induces B cell activation and Ab production, and preferentially activates memory B cells, where the virus persists [15,16]. The role of malaria infection in enhancing viral replication is further supported by our previous observation that acute malaria patients have high levels of Abs against the early viral lytic protein BZLF1 [14].

As a corollary of the in vitro experiments, it was important to analyze the impact of CIDR1α on PBMCs and tonsil B cells derived from healthy EBV-positive donors, and from patients with BL. As judged by quantification of EBV DNA, CIDR1α increased viral production. In healthy donors, the effect was more pronounced in tonsil B cells than in PBMCs. We believe that this observation reflects inherent differences in the frequency of memory B cells, as EBV-positive B cells are more frequent among tonsil B cells than in PBMC preparations [24].

EBV reactivation is suspected to play a role in BL lymphomagenesis and is indicated by the presence of high Ab titers to viral lytic components, which characteristically herald the onset of BL [11]. It has been previously suggested that BL may rise as a consequence of the combination of multiple mechanisms that involve chronic stimulation of the B cell compartment, increased viral production, and suppressed EBV-specific responses (reviewed by [28,29]). Our previous and present studies indicate that malaria has inherent effects on EBV–host balance that contribute to the abnormal viral load present in children from malaria-endemic areas and likely result in an increased risk for BL. In addition, the polyclonal B cell–activating capacity of malaria may increase the proliferation of EBV-positive cells [15]. The present data demonstrate that iRBCs and antigens related to the infectious cycle of malaria (such as CIDR1α) can trigger lytic production in EBV-carrying B cells. In vivo, the viral reactivation mediated by CIDR1α and other malaria-derived antigens might take place in the spleen, where the probability of trapped iRBCs to encounter EBV-carrying B cells is high, as over 40% of the splenocytes are B cells. Notably, splenomegaly is often observed in children living in highly endemic areas where BL occurs [30].

In conclusion, interactions between P. falciparum malaria and EBV could play a direct role in promoting the emergence of BL.

## Materials and Methods

### Cell lines.

Akata, a human BL-derived cell line carrying EBV, was maintained in RPMI 1640 (GIBCO-BRL, http://www.invitrogen.com) supplemented with 10% fetal bovine serum, 100 U/mL penicillin, and 2 mM glutamine, hereafter referred to as complete medium. The Akata GFP was previously generated by infecting the EBV-negative variant of Akata line with a recombinant EBV virus, carrying a cassette that encodes for both neomycin resistance gene under control of a thymidine kinase promoter and a modified GFP gene under control of the cytomegalovirus immediate early promoter [31]. With the recombinant EBV, the neomycin resistance gene and the modified GFP gene are inserted into the open reading frame of the non-essential EBV thymidine kinase gene *(BXLF1)* [31]. Akata cells were maintained in complete medium, and Akata-GFP cells in complete medium supplemented with 500 μg/ml geneticin (G418; Sigma-Aldrich, http://www.sigmaaldrich.com).

### Patients.

BL patients were recruited from the Ugandan Cancer Institute, Mulago Hill, Kampala, Uganda. Blood was collected from children with BL (range 4–7 y; mean age 5 y). BL was diagnosed on the basis of clinical symptoms, presence of a tumour mass, and histological analysis. The study protocol was approved by the higher degrees of the university, the research committee and ethical committee of Makerere University Faculty of Medicine, the Uganda National Council of Science and Technology, and the Karolinska Institutet Research Ethical Committee. Written, informed consent was obtained from guardians of study participants.

### Cell isolation.

Blood samples collected from two EBV-positive healthy volunteers (15–20 mL) and from BL patients (2–5 mL) before chemotherapy were processed the same day. PBMCs were isolated by centrifugation over Ficoll Plaque Plus (Amersham, http://www.amersham.com), washed in PBS, and resuspended in complete medium. Tonsils were obtained from patients undergoing routine tonsillectomy at Karolinska University Hospital in Stockholm, Sweden. Lymphocyte cell suspensions were prepared by mincing the tonsils and suspending the cells in complete medium. Isolated mononuclear cells were depleted of T cells by two rounds of rosette formation with ethyl isothiouronium bromide–treated sheep RBCs on ice. Rosettes were removed by centrifugation over lymphoprep (Nycomed Pharma, http://www.nycomed.com) [32]. The tonsil B cell purity was >95% as revealed by cytofluorimetric (FACS) analysis after staining with the pan–T cell Ab CD3 and the monocyte marker CD14.

### Parasites.

The highly rosetting and auto-agglutinating P. falciparum parasite clone FCR3S1.2 was obtained through cloning by micromanipulation of the mother clone FCR3 [33] and cultured according to standard methods. Parasites were maintained and expanded in RBCs (O Rh^+^) at 5% haematocrit. iRBCs were cultured in RPMI 1640 medium supplemented with 10% B+ human serum, 0,6% HEPES (GIBCO-BRL), 25 μg/mL gentamycin (GIBCO-BRL), 0,25% sodium bicarbonate (GIBCO-BRL), and 2 mM L-glutamine (GIBCO-BRL). Trophozoites were enriched by magnetic-assisted cell sorting, using a Vario-MACS (Miltenyi Biotec, http://www.miltenyibiotec.com). Synchronous P. falciparum parasite cultures, grown 24–28 h post-invasion, were washed twice in RPMI 1640 and resuspended in 5–10 mL of phosphate-buffered saline (PBS) with 2% bovine serum albumin (BSA). Rosettes were disrupted mechanically by repeated passage through a 0,6-mm-thick injection needle. The material was slowly added to a MACS separation column mounted in the magnet and rinsed with 50 mL of 2% BSA in PBS. The iRBCs were eluted in 50 mL of 2% BSA in PBS after removing the column from the magnet, spun down at 500*g,* and resuspended in 1 mL of RPMI 1640. Following enrichment, the parasitemia was evaluated by fluorescence microscopy after addition of one drop of acridine orange (10 μg/mL).

### Parasite extracts.

Parasite extracts were prepared as previously described [34]. Briefly, parasites at trophozoite stage (iRBCs, 28 h post-invasion) or RBCs were washed, resuspended in PBS, and sonicated (25w) on ice at short intervals for 2 min. The extracts were then centrifuged at 500*g* for 10 min at 4 °C and filter-sterilized. After determination of protein concentration by Bradford Assay (Bio-Rad, http://www.bio-rad.com), the extracts were diluted with PBS.

### Recombinant CIDR1α.

The sequence of the CIDR1α domain of the PfEMP1 from clone FCR3S1.2-var1 was optimized for codon adaptation in *Escherichia coli,* re-synthesized chemically (GeneArt, http://www.geneart.com), cloned into the pGEX4T-1 vector (Amersham Pharmacia, http://www.gelifesciences.com), and expressed in E. coli BL21 CodonPlus-RIL (Stratagene, http://www.stratagene.com) as fusion protein with the GST. Non-recombinant pGEX4T-1 was used to produce GST as control protein. Protein purification was carried out according to an optimized protocol [35], and the recombinant protein was purified on glutathione-sepharose column GSTrap FF (Amersham Pharmacia) according to the manufacturer's instructions. Throughout the paper, we refer to the recombinant CIDR1α-GST fusion protein as CIDR1α, while GST is referred to as control protein.

### Binding assays.

*Akata iRBCs:* Akata cells and enriched iRBCs (trophozoite stage) were stained with PKH67 (green) and PKH26 (red) (Sigma-Aldrich), respectively, according to manufacturer's instructions. The binding was evaluated by fluorescence microscopy after co-incubation in PBS at room temperature (RT) for 1 h at a 1:2 ratio (Akata:iRBC).

*Akata CIDR1α:* Cells incubated for 1 h at RT in PBS containing GST or CIDR1α (1 μM) were washed twice in PBS and incubated for 30 min at RT with anti-GST Abs (Sigma-Aldrich) diluted 1:500 in PBS. After two washes with PBS, anti-mouse IgG Alexa-488-conjugated Ab (Molecular Probes, http://probes.invitrogen.com) diluted 1:100 in PBS was added for 30 min at 4 °C. Cell binding was analyzed by fluorescence microscopy, and the fluorescence intensity was measured with a FACSCalibur flow cytometer and analyzed with Cell Quest Pro software (Becton Dickinson, http://www.bd.com).

### Stimulation assays.

Twenty-four hours before experiments, Akata and Akata-GFP cells were suspended at a concentration of 10^6^/mL in complete medium and complete medium containing G418 (500 μg/mL), respectively. Fresh PBMCs were washed with PBS and resuspended in complete medium at a concentration of 10^5^ cells per ml. The cells were then seeded in 96-well plates and cultured in medium alone or with increasing concentrations of CIDR1α, GST (range 0–4 μM, corresponding to 0–200 μg/mL), or with anti-Ig (10 μg/mL) (Jackson ImmunoResearch, http://www.jacksonimmuno.com). After 48 h, cells were harvested for analysis. All tests were set up in multiple replicates.

### EBV DNA detection by real-time PCR.

DNA was extracted from cells and supernatants using the QIAamp Blood kit (Qiagen, http://www.qiagen.com) according to manufacturer's instructions and eluted in 50 μL of DEPC-treated water (Ambion, http://www.ambion.com). Purity and DNA concentration were evaluated using a NanoDrop ND-1000 spectrophotometer (http://www.nanodrop.com). The PCR primers and probe used for the quantification of EBV genomes were selected from the LMP-1 gene as previously described [13,14]. The primers used were the EBV-LMP1 forward primer 5′-AAGGTCAAAGAACAAGGCCAAG-3′ and the EBV-LMP1 reverse primer 5′-GCATCGGAGTCGG-3′. The fluorogenic probe (PE Applied Biosystems, http://www.appliedbiosystems.com) was synthesized using a FAM reporter molecule attached to the 5′ end and a TAMRA quench-er linked to the 3′ end (5′-AGGAGCGTGTCCCCGTGGAGG-3′). A standard curve was prepared using serial dilutions of DNA derived from the EBV-positive BL line Namalwa that contains two copies of EBV genome per cell. Detection was performed using an ABI Prism 7700 Sequence detection System (PE Applied Biosystems). Briefly, cycling parameters were 50 °C for 2 min, 95 °C for 10 min, 45 cycles 95 °C for 15 s, and 60 °C for 1 min. The EBV DNA copy number was calculated as mean of triplicates.

### Cell cycle analysis.

Cells were washed twice in ice-cold PBS and resuspended in 100 μL of PBS containing 50 μg propidium iodide/mL and 0.1% (v/v) triton X-100. After incubation at 4 °C for 8 h, cell DNA analysis was performed by flow cytometry (FACSCalibur, Becton Dickinson) using Cell Quest Pro software (Becton Dickinson). *p*-Values ≤ 0,05 were regarded as statistically significant.

### Western blot analysis.

Expression of BZLF1, the early lytic antigen, was assessed using a specific mouse Ab (DAKO, http://www.dako.com). Total extracts from 10^6^ cells were separated in 10% SDS polyacrylamide gels, blotted onto nitrocellulose filters probed with 1:500 dilution of the DAKO monoclonal Ab for 1 h at RT, and then reacted with horseradish-peroxidase–labelled anti-mouse Abs (Amersham). Immunocomplexes were visualized by enhanced chemiluminescence according to the manufacturer's instructions (Amersham). To control for equal loading, the total protein concentration of each sample was checked using Bradford assay.

### Statistical analysis.

Computations were performed with the Prism 4 package (GraphPad Software, http://www.graphpad.com). For parametric data, differences between groups were analyzed with Student *t*-test; for nonparametric data, differences between groups were analyzed with the Wilcoxon rank sum test. Results are expressed as mean ± SEM, and unless otherwise stated, considered statistically significant at *p* < 0,05.

## Abbreviations

Ab: antibody
BCR: B cell receptor
BL: endemic Burkitt lymphoma
CIDR1α: cystein-rich inter-domain region 1α
EBV: Epstein–Barr virus
FACS: fluorescent-activated cell sorting
GFP: green fluorescent protein
GST: glutathione-S-transferase
Ig: immunoglobulin
iRBC: infected red blood cell
PBMC: peripheral blood mononuclear cell
PfEMP1: Plasmodium falciparum erythrocyte membrane protein 1
RBC: red blood cell
